# Clinical and Biological Correlates of Preoperative Cognitive Functioning of Glioma and Meningioma Patients

**DOI:** 10.1155/2020/2054859

**Published:** 2020-05-08

**Authors:** Aiste Pranckeviciene, Vytenis P. Deltuva, Arimantas Tamasauskas, Jurate Zegliene, Adomas Bunevicius

**Affiliations:** ^1^Neuroscience Institute, Lithuanian University of Health Sciences, Eiveniu str. 4, LT-50161, Lithuania; ^2^Hospital Of Lithuanian University of Health Sciences, Neurosurgery Department, Eiveniu str. 2, LT-50009 Kaunas, Lithuania

## Abstract

**Objectives:**

This study aimed to investigate the association of high-sensitivity C-reactive protein (hsCRP) and *N*-terminal pro-B-type natriuretic peptide (NT-proBNP) serum concentrations with cognitive functions of glioma and meningioma patients.

**Methods:**

177 brain tumor patients awaiting for brain tumor surgery participated in the study. Patients were assessed preoperatively, using neuropsychological tests for verbal memory, psychomotor speed, mental flexibility, and verbal fluency. The functional status of patients was evaluated using the Karnofsky Performance Index. Blood samples were drawn for evaluation of serum hsCRP and NT-proBNP concentrations upon hospital admission.

**Results:**

The highest NT-proBNP concentration was observed in meningioma patients. Glioma and meningioma patients did not differ in hsCRB concentration. Patients in the highest hsCRP tertile were older and more frequently reported cardiovascular comorbidity. Patients in the highest NT-proBNP tertile were older, more frequently with cardiovascular comorbidity, females, and diagnosed with a meningioma. hsCRP was significantly related to slower psychomotor speed in high-grade glioma patients (*rho* = 0.30, *p* < 0.05). In meningioma sample, NT-proBNP correlated with decreased psychomotor speed (*rho* = 0.38, *p* < 0.01), mental flexibility (*rho* = 0.33, *p* < 0.01), worse cumulative learning (*rho* = −0.27, *p* < 0.05), and delayed recall (*rho* = 0.30, *p* < 0.01). However, the relationship between the NT-proBNP and cognitive functions became nonsignificant when demographic and clinical covariates were included into analysis. Higher hsCRP concentration remained significantly related to slower psychomotor speed (*p* = 0.02) and worse mental flexibility (*p* = 0.05) in glioma patients, independently from demographic and clinical covariates. Preoperative cognitive functioning was also predicted by older age, gender, side and location of the tumor, and tumor malignancy, and general functional status of a patient.

**Conclusions:**

NT-proBNP was not associated with memory, language, and attention/executive cognitive domains of glioma and meningioma patients. Increased hsCRP was related to slower psychomotor speed and worse mental flexibility in glioma patients, indicating that inflammation processes are important for cognitive functioning in glial tumors.

## 1. Introduction

Cognitive decline is a common and devastating sequalae of brain tumors (BTs) [1]. Cognitive impairment is often already present at the time of BT diagnosis, continues to deteriorate with disease progression [2, 3], and is an independent predictor of impaired quality of life, worse health status, and shorter survival [4, 5]. A better understanding of the cognitive impairment of BT patients is important towards decreasing the burden of cognitive symptoms and improving the health status of BT patients.

Numerous tumor and treatment-related factors were implicated in the progressive cognitive decline of BT patients. BTs can infiltrate and/or displace normal brain tissue, increase intracranial pressure, cause seizures, and disrupt structural and functional brain connectivity, subsequently leading to cognitive decline [1, 6]. However, the level of global cognitive decline and impairment across more discrete cognitive domains usually does not linearly correlate with BT location, laterality, and volume [2, 7, 8]. There is significant inconsistency regarding the relationship between left/right side tumor location [9–11], histological diagnosis [12–14], and level of cognitive impairment across studies. These observations suggest that other nonstructural BT-related mechanisms might be important for the cognitive functioning of BT patients.

Inflammatory response is important for development, progression, and prognosis of malignant [15, 16] and benign [17, 18] BTs. Greater circulating hsCRP concentration was linked to shorter survival of glioma [19] and meningioma [20] patients. Studies in general population and in patients with non-CNS disorders have consistently reported that elevated hsCRP serum concentrations are associated with worse global cognitive performance, decreased memory function [21, 22], worse inductive reasoning and vocabulary [23], and changes in attentional-executive-psychomotor functions [24]. However, studies exploring the possible association of inflammatory response with the cognitive functioning of BT patients are lacking.

NT-proBNP is produced by cardiac myocytes in response to volume overload and is used for diagnosis and monitoring of heart failure patients [25]. Greater serum NT-proBNP concentrations were previously implicated in worse general cognitive functioning and increased risk of cognitive impairment [26, 27], decreased perception speed [28], and executive function [29] in community-dwelling adults and in cardiac patients. It was recently shown in BT patients that elevated NT-proBNP levels are associated with a greater five years mortality risk and greater global cognitive decline [25]. Therefore, the possible association between NT-proBNP and cognitive impairment of BT patients warrants further research.

In this study, we aimed to investigate the association of hsCRP and NT-proBNT serum concentrations with memory, language, and attention/executive cognitive domains of glioma and meningioma patients.

## 2. Materials and Methods

### 2.1. Procedure

The study protocol and consent procedure were approved by the Ethics Committee for Biomedical Research of the Lithuanian University of Health Sciences, Kaunas, Lithuania, and were in accordance with the 1964 Helsinki Declaration and its later amendments or comparable ethical standards. Written informed consent was obtained from each study patient before inclusion in the study.

Consecutive patients admitted at the Department of Neurosurgery of Hospital of Lithuanian University of Health Sciences, Kaunas, Lithuania, for glioma or meningioma surgery in a period from October 2015 to May 2017 were invited to participate in this cross-sectional observational cohort study. The study exclusion criteria included severe preoperative cognitive deficits and/or neurological impairment leading to inability to complete the study tasks, established diagnosis of acute infections (based on clinical symptoms and routine preoperative laboratory workup) or documented chronic inflammatory disorders (based on medical histories).

Blood samples were drawn for evaluation of serum hsCRP and NT-proBNP concentrations upon hospital admission. Neuropsychological assessment was performed from two to three days before BT surgery by a certified medical psychologist. Medical history, clinical characteristics, and functional status of the study patients were recorded by the study neurosurgeon. Histological BT diagnoses were verified from postoperative pathology reports. Information about tumor laterality and location was obtained by reviewing preoperative imaging (MRI or CT) studies.

### 2.2. Patients

During the study period, a total of 277 patients with histologically confirmed diagnoses of glioma or meningioma operated at our department were approached and invited to participate in the study. Three (1%) patients refused to participate and 14 (5%) patients were unable to complete the study tasks due to severe neurological/auditory impairment or poor health status. Blood samples were missing for 16 (6%) of patients. Sixty-seven (24.19%) patients were not evaluated for cognitive functioning due to logistic reasons that included shorter than three days of preoperative stay and intensive preparation for surgery schedule. Consequentially, the final study sample was comprised of 177 patients that included 90 (51%) glioma and 87 (49%) meningioma patients.

### 2.3. Study Instruments

Karnofsky performance index (KPI) was used for the assessment of functional status [30]. The KPI is an 11-point rating scale designed to measure a patient's ability to carry his/her normal activities and dependence on help and nursing care. The total KPI score ranges from 100 (normal functioning) to 0 (death).

The Hopkins Verbal Learning Test-Revised (HVLT-R) is a list of learning verbal memory task [31]. The test consists of 12 words that are read aloud for three trials, each trial followed by a patient's free recall. After approximately 20 min. delay, the patient is asked to recall the words. Two scores: cumulative learning (total number of words recalled in trial 1, 2, and 3), and delayed recall (number of words recalled after delay) are analysed in this study. Higher scores represent better verbal memory function.

Trail Making Test (TMT, Parts A and B) is a test for assessment of visual attention, psychomotor speed, mental flexibility, and executive functioning [32]. The task requires the patient to connect a sequence of 25 targets (numbers 1, 2, 3, etc. in Part A, and alternate between numbers and letters 1, A, 2, B, etc. in Part B) on a sheet of paper. Time (in seconds) to complete test tasks was recorded and used as an indicator of psychomotor speed and mental flexibility. Longer performance time represents slower psychomotor speed and worse mental flexibility.

Verbal Fluency Test consists of phonemic and semantic fluency tasks [33]. During phonemic fluency task patients are asked to produce as many words as possible beginning with a specific letter within one-minute interval. Three trials are recorded. For the assessment of semantic fluency, patients are asked to produce as many animal names as possible within one-minute interval. A total number of words produced during both tasks are used as verbal fluency indicator in this study; a higher number of words represents better verbal fluency.

### 2.4. Assessment of hsCRP and NT-proBNP Concentrations

Venous blood samples were centrifuged, and serum samples were stored frozen in -80°C. Serum concentrations of hsCRP and NT-proBNP were evaluated at the certified laboratory after the study completion. hsCRP concentration was evaluated using the Beckman Coulter Unicel DXC 600 kit. Serum concentrations of NT-proBNP were assessed using a radioimmunoassay method (Roche cobas analyzer; Roche Diagnostics, UK).

### 2.5. Data Analysis

Data was analysed using the IBM SPSS Statistics 20 package. Pearson chi-square criteria and nonparametric Kruskal-Wallis test were used for comparison of sociodemographic and clinical characteristics, and hsCRP and NT-proBNP concentrations between meningioma and glioma patients. Before proceeding with further analysis, the dataset was evaluated for outliers: univariate outliers of hsCRP and NT-proBNT and multivariate outliers of cognitive variables based on Mahalanobis distance were removed. Patients were divided into tertiles according to their hsCRP and NT-proBNP levels: for hsCRP: <0.59/0.59-1.40/>1.40 mg/l, for NT-proBNP: <54.10/54.10-163.77/>163.77 ng/l.

Pearson chi-square test and one-way ANOVA were used to compare sociodemographic and clinical variables stratified by hcCRP tertiles and NT-proBNP tertiles. Linear regression was used to analyse the relationship of hsCRP and NT-proBNT concentrations with scores on cognitive tests controlling for demographic and clinical variables. Regression analysis was performed for glioma and meningioma patients separately. Covariates used in the regression analysis were patient age (in years), gender (male -1, female -0), education status (less than 12 years-0, more than 12 years of education-1), preoperative functional status (KPI score), histological BT diagnosis (for glioma sample only, low-grade glioma-0, high-grade glioma-1), BT laterality (left or bilateral-1, right-0), and BT location (frontal and/or temporal -1, other -0).

## 3. Results

Demographic and clinical characteristics as a function of BT histological diagnosis are presented in Table 1. As expected, females were significantly overrepresented in meningioma sample (*p* = 0.03). When compared to glioma patients, meningioma patients were older (*p* < 0.01), were more likely to live alone (*p* = 0.03), have histories of cardiovascular diseases (*p* = 0.03), had bifrontal tumors, and had greater NT-proBNP concentrations. High-grade glioma patients had lower initial functional status when compared to low-grade glioma and meningioma patients (*p* < 0.01). Glioma and meningioma patients did not differ in education status, tumor location involving frontal/temporal lobes and hsCRP concentrations.

Patients in the highest hsCRP tertile were significantly older (*p* = 0.02) and more frequently reported cardiovascular diseases (*p* = 0.03) relative to patients in lower tertiles (Table 2). Patients in the highest NT-proBNP tertile were older (*p* = 0.04), more often reported cardiovascular diseases (*p* < 0.01), were females (*p* = 0.04) and were diagnosed with meningioma (*p* < 0.01) when compared to lower tertiles.

Initial correlation analysis revealed some weak but significant relationships between hsCRP and NT-proBNP concentrations and cognitive measures in brain tumor patients. hsCRP was significantly related to slower psychomotor speed in high-grade glioma patients (Spearman *rho* = 0.30, *p* < 0.05). In meningioma sample, NT-proBNP correlated with decreased psychomotor speed (Spearman *rho* = 0.38, *p* < 0.01), mental flexibility (Spearman *rho* = 0.33, *p* < 0.01), worse cumulative learning (Spearman *rho* = −0.27, *p* < 0.05), and delayed recall (Spearman *rho* = 0.30, *p* < 0.01). A similar but nonsignificant trend was observed in low-grade glioma patients: NT-proBNP correlated with slower psychomotor speed (Spearman *rho* = 0.32, *p* < 0.12) and mental flexibility (Spearman *rho* = 0.30, *p* = 0.15). However, the cognitive functioning of brain tumor patients depends on many factors, that is why linear regression analysis was used to compare cognitive performance in relation to hsCRP and NT-proBNP controlling for clinical and demographic covariates.

In glioma patients (Table 3), higher hsCRP concentration was significantly related to slower psychomotor speed (*p* = 0.02) and worse mental flexibility (*p* = 0.05), independently from demographic and clinical covariates. Older age was related to slower psychomotor speed (*p* < 0.001), decreased mental flexibility (*p* < 0.001), slower cumulative learning (*p* = 0.02), worse delayed recall (*p* < 0.001), and decreased verbal fluency (*p* = 0.01). The effect of gender was observed on cumulative learning (*p* = 0.03) and delayed recall (*p* < 0.001) with females demonstrating better results. The left or bifrontal location of the tumor was related to worse cumulative learning (*p* < 0.001), delayed recall (*p* < 0.001), and decreased verbal fluency (*p* < 0.001). Tumor grade was related to mental flexibility (*p* = 0.05) and cumulative learning (*p* = 0.01). Frontal and/or temporal tumor location was not related to cognitive functioning in glioma patients. Decreased functional status of the glioma patient was significantly related to decreased psychomotor speed (*p* < 0.001), mental flexibility (*p* -0.03), worse verbal memory performance (cumulative learning *p* = 0.03; delayed recall = 0.01). NT-proBNP was not related to the cognitive functioning of glioma patients, when demographic and clinical variables were included into analysis.

In meningioma patients, the only tendency was observed hsCRP to be related with slower psychomotor speed (*p* = 0.06) (Table 4). NT-proBNP was not related to cognitive functioning of meningioma patients. Cognitive functioning of meningioma patients was related to age, older patients demonstrated decreased mental flexibility (*p* < 0.001), worse verbal learning (*p* = 0.01), decreased delayed recall (*p* < 0.001), and verbal fluency (*p* = 0.02). Females when compared with males, demonstrated better verbal fluency (*p* = 0.01). Verbal fluency was also related to education (*p* = 0.05) and left/bifrontal location of the tumor (*p* = 0.02). Similarly to glioma patients, frontal and/or temporal tumor location of the tumor was not related to cognitive functioning of meningioma patients. Decreased functional status was significantly related to decreased psychomotor speed (*p* = 0.05), mental flexibility (*p* < 0.001), worse cumulative learning (*p* = 0.01), and decreased verbal fluency (*p* = 0.05).

## 4. Discussion

In this study, we aimed to investigate the possible association of hsCRP (proinflammatory biomarker) and NT-proBNP (cardiac impairment biomarker) with preoperative cognitive functioning of glioma and meningioma patients. Greater NT-proBNP serum concentration was associated with lower psychomotor speed, worse mental flexibility, less effective learning, and decreased delayed recall of verbal information of meningioma patients in univariate correlation analyses. A similar trend was observed in low-grade glioma patients. However, the association became nonsignificant after adjusting for demographic, clinical, and tumor-related variables, indicating that observed relationships were caused by other variables, most likely, presence of comorbid cardiovascular disease, but not NT-proBNP directly. Contrary, hsCRP correlated with slower psychomotor speed in high-grade glioma patients in univariate correlation analysis and this relationship remained significant in linear regression analysis when clinical and demographic factors were considered, indicating the unique impact of inflammation processes on cognitive functioning of glioma patients.

To our knowledge, only one previous study investigated the relationship between levels of NT-proBNP and cognitive functioning of BT patients. Bunevicius et al. found that greater NT-proBNP serum concentrations correlated with general cognitive decline in a mixed sample of BT patients [25]; however, this association was not adjusted for potential cofounders. Similarly, in a sample of stroke patient levels, NT-proBNP at admission was associated with worse cognitive functioning in univariate analysis, but the relationship became nonsignificant when the model was adjusted for age, stroke type, and other clinical variables [34]. On the other hand, in a sample of coronary artery disease patients, NT-proBNP remained significantly associated with slower psychomotor speed after many sociodemographic and clinical covariates were controlled [28]. These findings suggest that worse myocardial function of BT patients might be related with global cognitive impairment, while the predictive value for more subtle cognitive functioning is questionable. However, the results of the study should be interpreted with caution given a moderate sample size and heterogeneity in patient age and tumor characteristics.

Daniels et al. [29] hypothesized that the association between NT-proBNP levels and poor cognitive functioning may reflect the presence of underlying cardiovascular disease. The heart interplays with other organs and deterioration of cardiac function inevitably leads to other organ dysfunction, including the decreased function of the brain [35]. Although the complete mechanism of heart-brain interaction still needs to be developed, at least several heart disease-related factors may contribute to decreased brain functions [36]. Heart diseases may cause local disturbances in cerebral blood flow leading to complete and incomplete infarcts, and microbleeds [37, 38], white matter tract damage, grey matter loss, general decrease in cortical thickness, and functional brain changes [39], including changes in functional brain connectivity. Cardiovascular disorders are common but often underrecognized in BT patients [40, 41]. Indeed, in our sample, nearly one-third of meningioma patients and 22% of glioma patients presented with cardiovascular comorbidity. In line with previous findings, we found that cardiovascular comorbidity and older age were related to greater NT-proBNP concentrations [25, 26], suggesting that NT-proBNP can be a valuable biomarker of underlying cardiovascular function impairment of BT patients. However, Ruggieri et al. [18] in a sample of 108 BT patients found no association between increase in NT-proBNP and cardiac dysfunction; levels of NT-proBNP were directly associated with cerebral damage. Clinical importance of subclinical impairment of cardiac function as well as its clinical biomarkers warrants further research in BT patient population despite our preliminary negative results.

In our sample, hsCRP serum concentrations were statistically significantly associated with decreased psychomotor speed and mental flexibility in glioma patients. Numerous studies documented that greater proinflammation cytokine concentrations are associated with worse cognitive functioning [21, 23, 26, 27, 29, 42, 43]. Previous research showed that inflammation is associated with decreased hippocampal volume [21], impaired endothelial function of brain vasculature [44], neurodegeneration [29], and impaired neurogenesis [45], dysregulation of frontal neural pathways [43] or may have indirect impact on cognitive functioning via vascular factors [42]. It is known that inflammatory response is important for the development, progression, and survival of malignant gliomas [15, 16, 19]. However, the impact of inflammation on the cognitive functioning of glioma patients should not be overestimated; the direct local negative impact of BT on normal brain functioning potentially overwhelms more subtle effects of inflammatory response. For example, in our study, patients' functional status was one of the most consistent predictors of cognitive functioning both in glioma and meningioma patients. Further research should address the importance of proinflammatory response for long-term cognitive outcomes after BT resection, because the inflammation was linked to more aggressive BT behavior and brain capacity to recover [46, 47].

Worse performance on cognitive tests was associated with higher tumor grade, left-sided or bifrontal tumors, and worse general functional status. Campanella et al. [48] also reported that high-grade glioma patients had a worse preoperative cognitive functioning when compared to low-grade and meningioma patients. Several studies reported more profound cognitive impairment in patients with left-sided tumors [12, 49]. However, other studies did not find differences in cognitive functioning as a function of tumor location or type [1, 13, 50, 51]. Thus, further studies in larger and with more homogenous BT patient cohort(s) with regards to tumor lateralization and histological characteristics are encouraged.

Age was consistently related to worse cognitive functioning both in glioma and meningioma patients. These results are expected as it is known that cognitive functioning decreases during the life span, and this change significantly impacts neuropsychological tests performance [52, 53]. However, a recent review of Seblova et al. [54] showed that substantial heterogeneity in cognitive capacities in older age still exists that cannot be explained by age or education; thus, decreased cognitive performance should not be automatically attributed to older age only. Some of the processes that currently are considered as part of normal ageing, in fact might be a disease process; thus, our understanding of healthy ageing should be updated. Ageing in contexts of brain tumors and ability to recover BT decreased cognitive functioning in older age is still underresearched [55]; identification of factors that contributes to cognitive recovery in older patients might be an important research area.

Several limitations of the current study should be acknowledged. The sample size of low-grade glioma patients was small, and the distribution of bilateral tumors was different in glioma and meningioma patients. Histories of cardiovascular disorders and inflammatory disorders were based on medical histories and patient self-report and were not prospectively evaluated. Prospective assessment for concurrent health conditions that can directly impair the studied biomarkers should be considered in the future studies in order to discern possible confounding effect. On the other hand, reasonable sample size, prospective evaluation of cognitive functioning, and adjustment for potential confounders are strengths of this study.

## 5. Conclusions

NT-proBNP was not associated with memory, language, and attention/executive cognitive domains of glioma and meningioma patients. Increased hsCRP was related to slower psychomotor speed and worse mental flexibility only in glioma patients. Cognitive performance was also related to the functional status of patients, age, gender, side and location of the tumor, and tumor malignancy in case of glioma.

## Figures and Tables

**Table 1:** Characteristics of the study sample by tumor diagnosis.

		I-II grade glioma	III-IV grade glioma	Meningioma	*P*	Total
		Mean (SD) or *N* (%)				
Gender	Male	14 (56.0)	27 (41.5)	25 (28.7)	0.03	66 (37.3)
	Female	11 (44.0)	38 (58.5)	62 (71.3)		111 (62.7)

Age in years		38.5 (11.2)	55.1 (13.5)	63.1 (11.3)	<0.01	56.7 (14.6)

Marital status	Living alone	4 (16.0)	16 (24.6)	35 (40.2)	0.03	55 (31.1)
	With partner	21 (84.0)	49 (75.4)	52 (59.8)		122 (68.9)

Education	<=12 years	7 (28.0)	23 (35.4)	37 (42.5)	0.37	67 (37.9)
	>12 years	18 (72.0)	42 (64.6)	50 (57.5)		110 (62.1)

Cardiovascular disease	No	23 (92.0)	51 (78.5)	58 (66.7)	0.03	132 (74.6)
	Yes	2 (8.0)	14 (21.5)	29 (33.3)		45 (25.4)

Functional status (KPI score)		88.4 (10.43)	79.2 (16.3)	86.4 (10.7)	<0.01	84.1 (13.5)

Tumor side	Right	14 (56.0)	28 (43.1)	35 (40.2)	0.08	77 (43.5)
	Left	11 (44.0)	31 (47.7)	35 (40.2)		77 (43.5)
	Bifrontal	0 (0.0)	6(9.2)	17 (19.5)		23 (13.0)

Tumor location	Frontal and/or temporal lobes	20 (80.0)	50 (76.9)	66 (75.9)	0.91	136 (76.8)
	Other	5 (20.0)	15 (23.1)	21 (24.1)	0.35	41 (23.2)

hsCRP		1.5 (1.6) ml/l	2.5 (5.5) ml/l	4.0 (11.6) ml/l		3.1 (8.8) ml/l

NT-proBNP		60.6 (83.0) nl/l	157.9 (222.4) nl/l	328.0 (717.8) nl/l	<0.01	227.7 (530.6) nl/l

**Table 2:** Characteristics of the study sample by hsCRP and NT-proBNP tertiles.

	High-sensitivity C-reactive protein in tertiles			p	N-terminal pro-B-type natriuretic peptide in tertiles			p
	Lowest <0.59 ml/l	Middle 0.59-1.40 ml/l	Highest >1.40 ml/l		Lowest <54.10 nl/l	Middle 54.10-163.77 nl/l	Highest >163.77 nl/l	
Gender								
Male	26 (39.4)	18 (27.3)	22 (32.2)	0.15	29 (44.6)	19 (29.2)	17 (26.2)	0.04
Female	31 (29.2)	44 (41.5)	31 (29.2)		28 (26.7)	39 (36.4)	40 (37.4)	
Age								
Age in years	52.3 (14.6)	57.4 (14.4)	59.5 (13.5)	0.02	46.1 (13.1)	57.8 (12.2)	64.3 (11.5)	<0.01
Marital status								
Living alone	14 (27.5)	22 (43.1)	15 (29.4)	0.41	13 (24.5)	19 (35.8)	21 (39.6)	0.25
With partner	43 (35.5)	40 (33.1)	38 (31.4)		44 (37.0)	39 (32.8)	36 (30.3)	
Education								
<=12 years	21 (31.8)	24 (36.4)	21 (31.8)	0.95	19 (29.7)	21 (32.8)	24 (37.5)	0.61
>12 years	36 (34.0)	38 (35.8)	32 (30.2)		38 (35.2)	37 (34.3)	33 (30.6)	
Cardiovascular disease								
No	49 (38.0)	46 (35.7)	34 (26.4)	0.03	50 (38.2)	48 (36.6)	33 (25.2)	<0.01
Yes	8 (18.6)	16 (37.2)	19 (44.2)		7 (17.1)	10 (24.4)	24 (58.5)	
Functional status								
KPI	83.9 (13.2)	85.2 (10.9)	86.7 (9.2)	0.43	86.9 (10.0)	84.5 (10.6)	84.6 (12.4)	0.43
Tumor side								
Right	22 (28.9)	31 (40.8)	23 (30.3)	0.07	28 (36.4)	25 (32.5)	24 (31.2)	0.56
Left	32 (42.7)	20 (26.7)	23 (30.7)		25 (34.2)	25 (34.2)	23 (31.5)	
Bifrontal	3 (14.3)	11 (52.4)	7 (33.3)		4 (18.2)	8 (36.4)	10 (45.5)	
Tumor location								
Involving frontal and/or temporal lobes	47 (35.3)	44 (33.1)	42 (31.6)	0.30	48 (36.4)	43 (32.6)	41 (31.1)	0.25
Other	10 (25.6)	18 (46.2)	11 (28.2)		9 (22.5)	15 (37.5)	16 (40.0)	
Type of the tumor								
I-II grade glioma	10 (40.0)	6 (24.0)	9 (36.0)	0.55	17 (68.0)	6 (24.0)	2 (8.0)	<0.01
III-IV grade glioma	23 (35.9)	23 (35.9)	18 (28.1)		21 (32.3)	22 (33.8)	22 (33.8)	
Meningioma	24 (28.9)	33 (39.8)	26 (31.3)		19 (23.2)	30 (36.6)	33 (40.2)	

**Table 3:** Summary of linear regression results. Relationships between hsCRB, NT-proBNP, and cognitive functioning in glioma patients in contexts of demographic and clinical variables.

	Psychomotor speed *F* (9, 80) = 6.51, *p* < 0.001			Mental flexibility *F* (9, 80) = 6.56, *p* < 0.001			Cumulative learning *F* (9, 80) = 6.58, *p* < 0.001			Delayed recall *F* (9, 79) = 9.29, *p* < 0.001			Verbal fluency *F* (9, 79) = 4.84, *p* < 0.001		
	*B*	95.0% CI	*t*	*B*	95.0% CI	*t*	*B*	95.0% CI	*t*	*B*	95.0% CI	*t*	*B*	95.0% CI	*t*
hsCRB	3.90	(0.61, 7.20)	2.36∗	3.54	(0.02, 7.07)	2.00∗	-0.01	(-0.25, 0.23)	-0.10	-0.10	(-0.22, 0.03)	-1.58	-0.26	(-1.02, 0.50)	-0.68
NT-proNBP	-0.05	(-0.14, 0.03)	-1.20	-0.05	(-0.14, 0.04)	-1.14	0.00	(-0.01, 0.01)	0.27	0.00	(0.00, 0.00)	-0.03	0.01	(-0.01, 0.03)	0.81
Gender	13.12	(-17.79, 44.03)	0.85	20.43	(-12.62, 53.47)	1.23	-2.46	(-4.72, -0.20)	-2.17∗	-1.69	(-2.84, -0.54)	-2.93∗∗	-4.28	(-11.40, 2.84)	-1.20
Age	2.04	(0.73, 3.36)	3.09∗∗	2.47	(1.06, 3.88)	3.49∗∗	-0.12	(-0.22, -0.02)	-2.45∗	-0.10	(-0.15, -0.06)	-4.26∗∗	-0.43	(-0.74, -0.13)	-2.84∗
Education	1.28	(-31.06, 33.62)	0.08	3.83	(-30.74, 38.41)	0.22	0.24	(-2.12, 2.61)	0.21	0.09	(-1.10, 1.28)	0.15	1.82	(-5.64, 9.28)	0.49
Malignancy	11.73	(-25.85, 49.30)	0.62	39.90	(-0.27, 80.07)	1.98∗	-3.55	(-6.29, -0.80)	-2.57∗	-0.57	(-1.96, 0.82)	-0.82	-6.67	(-15.33, 1.98)	-1.54
Left/bifrontal localization	5.38	(-23.86, 34.61)	0.37	11.97	(-19.28, 43.22)	0.76	-3.68	(-5.81, -1.54)	-3.43∗∗	-2.26	(-3.34, -1.18)	-4.17∗∗	-10.52	(-17.32, -3.72)	-3.08∗∗
Frontal and/or temporal location	-21.31	(-59.32, 16.69)	-1.12	-6.26	(-46.89, 34.37)	-0.31	0.38	(-2.39, 3.16)	0.28	-0.56	(-1.96, 0.84)	-0.80	-6.33	(-15.10, 2.45)	-1.44
Functional status (KPI)	-1.88	(-2.92, -0.84)	-3.59∗∗	-1.24	(-2.36, -0.13)	-2.23∗	0.09	(0.01, 0.16)	2.23∗	0.05	(0.02, 0.09)	2.77∗	0.20	(-0.05, 0.45)	1.57

**Table 4:** Summary of linear regression results. Relationships between hsCRB, NT-proBNP, and cognitive functioning in meningioma patients in contexts of demographic and clinical variables.

	Psychomotor speed *F* (8, 78) = 2.38, *p* < 0.05			Mental flexibility *F* (8, 76) = 5.69, *p* < 0.001			Cumulative learning *F* (8, 78) = 5.23, *p* < 0.001			Delayed recall *F* (8, 78) = 3.86, *p* < 0.001			Verbal fluency *F* (8, 78) = 4.54, *p* < 0.001		
	*B*	95.0% CI	*t*	*B*	95.0% CI	*t*	*B*	95.0% CI	*t*	*B*	95.0% CI	*t*	*B*	95.0% CI	*t*
hsCRB	1.18	(-0.03, 2.39)	1.94^†^	0.61	(-0.71, 1.93)	0.92	-0.04	(-0.12, 0.04)	-0.96	-0.01	(-0.06, 0.04)	-0.39	-0.12	(-0.40, 0.16)	-0.87
NT-proNBP	-0.01	(-0.03, 0.02)	-0.45	-0.01	(-0.03, 0.01)	-1.05	0.00	(0.00, 0.00)	-1.52	0.00	(0.00, 0.00)	-1.70	0.00	(0.00, 0.01)	0.60
Gender	15.08	(-14.18, 44.34)	1.03	7.01	(-25.01, 39.03)	0.44	-1.56	(-3.51, 0.40)	-1.59	-0.50	(-1.79, 0.79)	-0.77	-9.38	(-16.11, -2.66)	-2.78^∗^
Age	0.94	(-0.37, 2.24	1.43	2.84	(1.41, 4.27)	3.96∗∗	-0.13	(-0.22, -0.04)	-2.93∗	-0.09	(-0.15, -0.04)	-3.25∗∗	-0.37	(-0.67, -0.07)	-2.48∗
Education	-17.29	(-44.84, 10.26)	-1.25	-27.29	(-57.59, 3.02)	-1.79	1.47	(-0.37, 3.30)	1.59	-0.17	(-1.39, 1.04)	-0.29	6.46	(0.12, 12.79)	2.03∗
Left/bifrontal localization	17.93	(-9.48, 45.35)	1.30	21.42	(-8.63, 51.48)	1.42	-0.94	(-2.77, 0.89)	-1.02	-0.57	(-1.78, 0.64)	-0.94	-7.27	(-13.57, -0.97)	-2.30∗
Frontal and/or temporal location	7.96	(-23.19, 39.11)	0.51	4.27	(-29.74, 38.29)	0.25	1.02	(-1.06, 3.10)	0.98	-0.66	(-2.03, 0.72)	-0.95	-1.13	(-8.29, 6.03)	-0.31
Functional status (KPI)	-1.28	(-2.55, -0.01)	-2.00∗	-2.15	(-3.54, -0.76)	-3.08∗∗	0.12	(0.03, 0.20)	2.74∗	0.04	(-0.01, 0.10)	1.50	0.30	(0.01, 0.59)	2.03∗

## Data Availability

The data used to support the findings of this study are available from the corresponding author upon request.
